# A novel method for detecting extra-home range movements (EHRMs) by animals and recommendations for future EHRM studies

**DOI:** 10.1371/journal.pone.0242328

**Published:** 2020-11-30

**Authors:** Todd C. Jacobsen, Kevyn H. Wiskirchen, Stephen S. Ditchkoff

**Affiliations:** School of Forestry and Wildlife Sciences, Auburn University, Auburn, Alabama, United States of America; US Geological Survey, UNITED STATES

## Abstract

Infrequent, long-distance animal movements outside of typical home range areas provide useful insights into resource acquisition, gene flow, and disease transmission within the fields of conservation and wildlife management, yet understanding of these movements is still limited across taxa. To detect these extra-home range movements (EHRMs) in spatial relocation datasets, most previous studies compare relocation points against fixed spatial and temporal bounds, typified by seasonal home ranges (referred to here as the “Fixed-Period” method). However, utilizing home ranges modelled over fixed time periods to detect EHRMs within those periods likely results in many EHRMs going undocumented, particularly when an animal’s space use changes within that period of time. To address this, we propose a novel, “Moving-Window” method of detecting EHRMs through an iterative process, comparing each day’s relocation data to the preceding period of space use only. We compared the number and characteristics of EHRM detections by both the Moving-Window and Fixed-Period methods using GPS relocations from 33 white-tailed deer (*Odocoileus virginianus*) in Alabama, USA. The Moving-Window method detected 1.5 times as many EHRMs as the Fixed-Period method and identified 120 unique movements that were undetected by the Fixed-Period method, including some movements that extended nearly 5 km outside of home range boundaries. Additionally, we utilized our EHRM dataset to highlight and evaluate potential sources of variation in EHRM summary statistics stemming from differences in definition criteria among previous EHRM literature. We found that this spectrum of criteria identified between 15.6% and 100.0% of the EHRMs within our dataset. We conclude that variability in terminology and definition criteria previously used for EHRM detection hinders useful comparisons between studies. The Moving-Window approach to EHRM detection introduced here, along with proposed methodology guidelines for future EHRM studies, should allow researchers to better investigate and understand these behaviors across a variety of taxa.

## Introduction

A thorough understanding of animal space use across a landscape is necessary to effectively implement fundamental principles of conservation biology and wildlife management. Space use metrics such as home range size, home range fidelity, activity rate, dispersal distance, and path tortuosity can be used to draw inferences concerning resource selection and habitat use [1], predator-prey interactions [2, 3], disease epidemiology [4], bio-climatic relationships [5], and the effects of anthropogenic factors on wildlife populations [6, 7]. In turn, these inferences can provide a basis for biologically-driven management efforts such as preservation of key travel or migration corridors [8], controlling the spread of wildlife and livestock diseases [9, 10], reintroduction and translocation efforts [11, 12], protection of threatened species [13, 14], or adjustments to hunting regulations and seasons [15, 16].

The methodology with which researchers generate and analyze space use metrics is constantly evolving to allow for more accurate, precise, and informative measurements. On a larger scale, the advancement of these methodologies helps researchers achieve a greater understanding of animal behaviors [17]. For example, the emergence of fine-scale GPS tracking technology has refined both the spatial and temporal scales at which animal relocations can be measured [17, 18]. Likewise, increased statistical software capabilities have facilitated the manipulation and analysis of spatial data which previously was cumbersome or infeasible, or only provided coarse-scaled information [19–21]. Advancements in methodology have also occurred through the emergence of home range estimators that incorporate time, variance in Brownian motion, or other animal movement parameters into their models and now result in more representative utilization distributions that reflect associated movements across a landscape, rather than independent relocation points (e.g., Brownian and dynamic Brownian bridge movement models, movement kernel density estimators [22–25]).

In his early work on home ranges, Burt [26] restricted his definition of the “home range” to include “that area traversed by the individual in its normal activities of food gathering, mating, and caring for young,” but felt that “Occasional sallies outside the area, perhaps exploratory in nature, should not be considered as in [*sic*] part of the home range.” Nearly 60 years later, Powell [27] agreed that the home range of an animal encompassed its day-to-day activities, but also acknowledged that Burt’s original definition provided little direction on “how to quantify occasional sallies or how to define the area from which sallies are made.” This latter question of defining an individual’s home range boundary to include regular movements but to exclude the infrequent outlying relocation points has since received considerable attention both in theory and mathematically (e.g., [28–30]), since it pertains more broadly to the general objective of defining an individual’s home range boundary, but the outlying relocation points themselves were for a long time widely ignored. However, as GPS-tracking technology has become capable of fine-scale temporal and spatial monitoring of animal movements, attention has been drawn to these irregular, long-distance, extra-home range movements (EHRMs)—sometimes referred to as “sallies” or, more often, “excursions.” This EHRM term, while not used in previous literature, is introduced here to serve as a common term encompassing all synonymous language utilized in other studies that describe animal movements extending outside of home range boundaries. A relatively small portion of behavioral and wildlife ecology studies across a varied list of taxa have investigated such movements (e.g., [31–50]), and scientific understanding of these movements is still inadequate.

To separate and quantify these “occasional sallies” from “that area traversed by the individual in its normal activities,” the fundamental questions at the forefront of EHRM-related studies that need to be answered are “What is the animal’s home range?” followed by “Did the animal leave its home range?” To determine this, most previous studies investigating EHRMs have employed a common methodology (hereafter referred to as the “Fixed-Period” method) of constructing temporally-fixed home range periods (e.g., seasonal or annual) from an animal’s collected relocation dataset to define its home range area, and then examining individual relocation datapoints within that temporally-fixed period to identify movements that fall outside of the associated home range. However, we posit that while this approach—that is, defining a temporally-fixed home range for the purpose of detecting EHRMs occurring within that same temporal period—can adequately detect some EHRMs undertaken by an animal, it can in many cases overlook EHRMs as well. This is particularly the case when an EHRM results in, or is at least followed by, a change in space use following that EHRM, such as when the area visited during the EHRM later becomes utilized with regularity and incorporated into that animal’s seasonal or annual home range. This point requires us to revisit the previous questions, “What is the animal’s home range?” and “Did the animal leave its home range?” and the specific verbiage used to phrase these questions.

An obvious and unstated tenet of animal home range research and analyses is that the summarized and delineated home range for an animal is a post-hoc analysis of that animal’s past, and documented, space use. Rarely would relocation data be used to describe or draw inferences about an animal’s behavior and space use before or after the period from which relocation data were collected. Additionally, while we can deduce that an animal’s space use at a given point in time is based to some degree on the individual’s cognitive map and past experiences related to the area [51–53], it is impossible to know the complete extent of its lifetime home range unless relocation data have been collected for the animal since birth. However, this point becomes more muddled in light of EHRM detection and analysis, as EHRMs represent temporally-specific snapshots of interest within a greater period of collected data. As highlighted by Powell and Mitchell [54], “To understand the mechanistic, biological foundations of home-range behavior, therefore, the estimated home range of an animal must be linked explicitly to its cognitive map.” This cognitive spatial map, which may evolve over time as an individual interacts with its environment, does not project into the future for an individual beyond the “current” point in time when that individual is experiencing its environment (though these cumulative cognitive maps no doubt influence future space use). Despite the fact that an animal’s comprehensive movement data may be available for examination to the researcher, at the instant at which the animal exhibits a behavior such as undertaking an EHRM (time *Y*), the animal has yet to experience those decisions, interactions, movements, and space-use which occur subsequent to time *Y*. Returning to the original two questions necessary for EHRM detection with this in mind, we propose that “What is the animal’s home range?” could be more appropriately stated as “What is the animal’s home range over the period from time *X* up to time *Y*?”, and “Did the animal leave its home range?” as “At time *Y*, did the animal leave its home range which existed from time *X* up to time *Y*?” Reframing the questions in this manner would help direct researchers away from utilizing home range data collected after an EHRM occurred to define the home range area from which the animal may be venturing.

To address this, we introduce an alternative, moving-window approach for detecting EHRMs that may shed additional light on factors influencing these behaviors. We propose a method for EHRM detection (hereafter, the “Moving-Window” method) that defines an animal’s home range based solely on the movements and space-use from a specified point in time and leading up to the given moment in time at which an EHRM occurs (time *Y*), rather than using relocations both before and after the EHRM to construct the home range which would then be used for EHRM detection. This approach additionally allows for further inquiry and investigation to determine whether EHRMs do, in fact, lead to subsequent shifts in space use and why this may occur. One of our primary objectives for this research was to introduce the novel Moving-Window approach as well as to compare the number and characteristics of EHRM detections between the Moving-Window and Fixed-Period methods using a dataset of white-tailed deer (*Odocoileus virginianus*) relocations from Alabama, USA as a proxy for species with regular home ranges.

Another primary objective of this research is to highlight how variability in methodologies among previous EHRM studies may further be hindering scientific understanding of these unique movements. Despite the influx of EHRM-related studies in the 21^st^ century, a clear set of definition criteria for these movements has not been established by researchers. This is evidenced by the myriad different terms used throughout the literature (e.g., “excursion,” “foray,” “sally,” and “exploratory movement”). Furthermore, researchers have not reached a general scientific consensus as to what, biologically or quantitatively speaking, constitutes an EHRM other than being described as “a long-distance movement.” This lack of consensus exists among studies examining EHRMs within and across taxa (e.g., mammalian carnivores [32, 37, 40, 42, 44], rodents [31, 41], reptiles [34, 38], avian species [35, 43, 49], and fish [33, 39]), and even among closely related species (e.g., white-tailed deer [55–59], roe deer, *Capreolus capreolus* [60, 61], and red deer, *Cervus elaphus* [62]). Comparative biological inferences and replicability of study design and findings are complicated by this variability. To demonstrate how this spectrum of definition criteria produces substantially different results and leads to variable interpretations of EHRM behaviors, we applied a suite of literature-derived EHRM definition criteria to a sample dataset of EHRMs from GPS-collared white-tailed deer. Though these analyses of EHRM detection methods, criteria, and terminology primarily utilize data and studies from white-tailed deer, our methods and conclusions relating to EHRMs can be applied broadly to a variety of taxa.

## Materials and methods

### Study area

We collected movement data for white-tailed deer from 4 study areas across Alabama, USA. Two of the areas—the Barbour Wildlife Management Area (31°59.73 N, 85°27.57 W) and the Oakmulgee Wildlife Management Area (32°57.39 N, 87°27.60 W)—were state-designated public wildlife management areas (WMAs). The Alabama Department of Conservation and Natural Resources granted permission for use of these study sites in our research. The Barbour WMA, located in southeastern Alabama, encompassed approximately 114 km^2^ of public land. This WMA contained a mixture of habitat types including longleaf pine (*Pinus palustris*) in various stages of regeneration, loblolly pine (*Pinus taeda*), mixed hardwood upland areas, and mixed hardwood bottoms. The Oakmulgee WMA, located in west-central Alabama, occupied the southern reaches of the Appalachian foothills and encompassed approximately 180 km^2^ of public land. Dominant vegetation included mature longleaf pine stands and widespread mature hardwood forests on upper ridges and slopes of the WMA, as well as mature hardwood bottomlands.

The third study area was a private commercial timber production area in Pickens County, AL (33°12.45 N, 87°52.01 W), and comprised 16 leasable hunting tracts collectively managed by timber company wildlife biologists. We received permission from the owner of the property for use of this study site. The Pickens County site encompassed approximately 49 km^2^ of loblolly pine in various stages of regeneration, as well as several post-harvest clearings. The fourth study area was located in Marengo County, AL (32°14.08 N, 87°51.11 W), and encompassed approximately 31 km^2^ of land. The Marengo County site consisted of 3 adjoining private landholdings, as well as adjacent private commercial timber production areas and hunting leases held by private landowners. Permissions were given to us by the property owners for use of these private landholdings as a study site in our research. This area was characterized by various stages of loblolly pine regeneration and mixed-age hardwood stands.

### Capture and handling

During the summer of 2014, we captured 30 deer (≥1 year old, both sexes) across the 4 study areas, and we captured an additional 8 deer during the summer of 2015 to replace those that died from natural or hunting-related causes over the course of the first year of data collection. We captured deer with the use of tranquilizer dart rifles (Pneu-Dart, Inc., Williamsport, Pennsylvania, USA) equipped with night vision riflescopes (Yukon Advanced Optics Worldwide, Inc., Vilnius, Lithuania) from elevated stands. We immobilized deer via intramuscular injection by a 2cc radio-transmitter dart (Pneu-Dart, Inc., Williamsport, Pennsylvania, USA) containing a Telazol and xylazine-hydrochloride mixture (125mg/ml Telazol administered at 4.0 mg/kg; Fort Dodge Animal Health, Fort Dodge, Iowa, USA; 100mg/ml xylazine-hydrochloride administered at 2.0 mg/kg; Lloyd Laboratories, Shenandoah, Iowa, USA).

We fitted all deer with a G2110D programmed-release global positioning system (GPS) collar (Advanced Telemetry Systems, Inc., Insanti, Minnesota, USA) and equipped them with two plastic ear tags, one per ear (Y-Tex Corporation, Cody, Wyoming, USA). The GPS collars were fluorescent orange in color and ear tags were bright yellow to aid hunters in identification of GPS-collared deer. We asked hunters on all study areas not to harvest GPS-collared deer [63]. Once collaring was complete, we reverse-immobilized deer via intramuscular injection of the antagonist Tolazine (100mg/ml tolazoline hydrochloride administered at 2.0 mg/kg, Lloyd Laboratories). After administration of the Tolazine reversal, we monitored all deer until they were completely ambulatory and indicating full awareness of their surroundings. Animal-handling procedures were approved by the Auburn University Institutional Animal Care and Use Committee (PRN# 2013–2323), and both capture and handling were conducted in accordance with the guidelines of the American Society of Mammalogists [64].

### Data collection and manipulation

GPS collars began collecting data from the time of deployment until the programmed, automatic collar release date during March–April 2016. We programmed GPS collars to obtain fixes every 3.5 hours from April 1 –September 30 and every 1 hour from October 1 –March 31. We selected these fix rates to maximize collar battery life over two successive breeding periods for each deer while still maintaining fine-scale data throughout the year, with more frequent fixes recorded during the period that encompassed pre-breeding, breeding, and post-breeding activities. Field tests to determine mean locational error of G2110D GPS collars in a similar ecosystem in the region were previously conducted and mean locational error was 12.9 m (SD = 9.8 m) in that study [65]. We employed a slightly more conservative, 15-m collar location error rate for data analysis throughout our study. We censored data for accuracy by removing 2-dimensional fixes with horizontal dilution of precision (HDOP) >5 and 3-dimensional fixes with position dilution of precision (PDOP) >10 or HDOP >6 [66, 67]. Additionally, we censored all data from the study that were obtained within 7 days post-capture to avoid any potential effects of capture on deer movement.

### Extra-home range movement detection

We identified and analyzed EHRMs from GPS relocations for each deer. We compared two different approaches for this analysis: the “Fixed-Period” method, and the “Moving-Window” method. All EHRMs detected by each method—including unique EHRMs (those detected by one method but not by the other) as well as paired EHRMs (those individual EHRMs detected by both methods) were manually inspected, and unique versus paired classification was determined based on EHRM start and end points as well as on EHRM characteristics and relocation points comprising EHRM movements.

#### Fixed-Period method

The first approach in our analysis implemented the temporally-fixed, seasonal home range method (Fixed-Period) frequently used for detecting EHRMs in other studies [55, 57, 59, 68, 69]. While the majority of past studies implementing this technique modelled temporally-fixed home ranges with seasonal home ranges, we recognize that multi-season and annual periods have also been employed in past EHRM analyses to define home range areas [70, 71]. For detection of EHRMs using the Fixed-Period method, we separated location data into 3 seasons based on general vegetative and climatic changes that occur throughout the year in south-central Alabama: spring (1 March– 30 June; 121 days), summer (1 July– 31 October; 122 days), and fall/winter (1 November– 28 February; 119 days). For each deer, we modelled seasonal home ranges if at least 60 days of relocation data were available within a season. We selected a period of 60 days to identify a representative area of use for deer at a local landscape level. This period duration adequately captured the consistent climatic and vegetative growth characteristics typical to the mild, southern climate of our study sites and was sufficiently representative of the full season lengths that we defined here. Therefore, seasonal home ranges modelled here varied between 60 days to the full season length. If <60 days remained before the end of the season in which capture occurred, our analysis began at the onset of the following season. In instances where >60 days of data were available in a given season but data were not collected for the entirety of that season (e.g., collar release, collar malfunction, deer mortality), we constructed a partial seasonal home range. We modelled all home ranges for our study using Brownian bridge movement models (BBMM, [23]) in R 3.1.1 (www.r-project.org). We summarized these home ranges at the 95% contour level and used a grid cell size of 100 m to map the utilization distribution. Though Powell [27], in his explanation of home range theory, indicated that the 95% contour holds no fundamental biological relevance, he did indicate that this value is widely accepted for use in home range studies and is a likely value to spatially eliminate occasional “sallies” in a relocation dataset. This exclusion of “sallies” (EHRMs) from the home range contour was the object of our analyses, and the standardization of the 95% contour across previous animal home range studies, particularly for the purpose of isolating EHRMs, further reinforced our selection of this probability value [29, 72].

We defined EHRMs as sequential strings of 3 or more GPS locations with at least 1 location extending ≥0.5 km outside of the 95% seasonal home range contour. Each EHRM started with the last point inside the home range contour before the individual left the home range and ended with the first point back inside the home range contour at the end of the EHRM. We employed a conservative distance of 0.5 km to balance capturing all purposeful EHRMs on a scale typical for white-tailed deer that have been identified in past EHRM analyses (0–8.0 km) [55–57, 59, 69], while also attempting to limit the inclusion of subtle range expansions and normal variability in daily movements along the periphery of the home range which necessarily fall outside of an estimated 95% contour (but that might be considered part of the home range if the home range contour was delineated at the 96% level, for example). We identified movements that met these criteria using an algorithm written in R (S1 File) and then visually verified each detected EHRM in ArcMap 10.2 (Environmental Systems Research Institute, Inc., Redlands, California, USA). We determined maximum travel distance for an EHRM as the Euclidian distance from the nearest edge of a 95% home range contour to the furthest point along that EHRM and defined EHRM duration as the elapsed time between the last fix inside the 95% home range contour prior to the start of the EHRM until the first point returning inside the home range contour at the end of the movement. We did not establish a minimum duration criterion for defining EHRMs; however, during our most intense fix-interval sampling period (1 fix/hour), the minimum duration possible was 2 hours, and minimum duration possible during our coarser fix-interval sampling period (1 fix/3.5 hours) was 7 hours.

#### Moving-Window method

Similar to our application of the Fixed-Period method, we constructed all home ranges for the Moving-Window method in R using a BBMM (95% contour, 100-m grid cell size) and again defined EHRMs as sequential strings of 3 or more GPS locations with at least 1 location extending ≥0.5 km outside of the 95% home range contour. However, rather than using fixed, seasonal home range periods as the basis for our home range models, this method utilized an iterative, shifting home range period. The fundamental purpose of the Moving-Window method was to allow for EHRM detection on each day of GPS-collar data when compared against the home range utilized over a preceding period of time leading up to the EHRM. In this manner, the home range used to detect EHRMs was only comprised of spatial data collected prior to the EHRM that was being examined, rather than spatial data occurring both before and after an EHRM (as in the Fixed-Period method). For each deer, beginning on the 8^th^ day post-capture and post-initiation of data collection (day 1), we constructed a 60-day, pre-EHRM home range (PreHR) spanning from day 1 to day 60. We selected a 60-day PreHR for this study for analysis and illustration purposes only; however, similar to our justification for the use of a 60-day minimum period for modelling a home range with the Fixed-Period method, a PreHR period of 60 days was selected because it was short enough to adequately capture most environmental resource availability shifts or internal physiological changes that may have influenced home range establishment and resource use, and yet long enough to identify a representative, typical area of space use for our study species. A period of 60 days captured the general climatic and vegetative growth characteristics of our study sites, given the mild, southern climate where this study occurred. Additionally, implementing a period of 60 days—versus a shorter frame such as a 30-day period—was more conducive to modeling of home range via a BBMM, given the frequency of our collar relocation interval and the subsequent number of overall data points. It should be noted that the specific length of time we used to construct the PreHR period is not a critical function of the Moving-Window method; the length of time selected for the PreHR can, and should, be determined based on the question of interest, study species and species biology, climatic and vegetative characteristics of study sites, as well as the fix rate interval and the home range estimator used.

**Fig 1:**
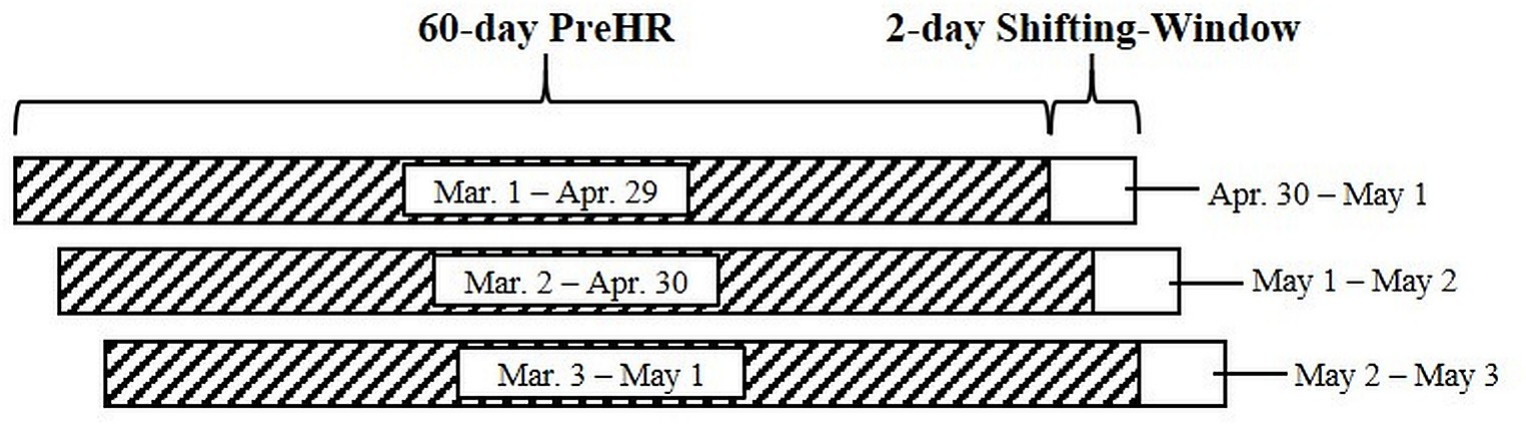
The Moving-Window method. Three example iterations of the Moving-Window method indicating the 60-day PreHR and the 2-day Moving-Window (not to scale). Each iteration shifts the PreHR and the Moving-Window by one day to examine for EHRMs on each subsequent day of relocation data.

Immediately following the 60-day PreHR period, a 2-day window (spanning day 61 to day 62) was examined by the algorithm for EHRMs using the criteria previously described. We then repeated this process iteratively, advancing one day forward per iteration, so that each PreHR began on day *i* (where *i* is the iteration number) and lasted until day *i* + 59, with a subsequent 2-day window spanning from day *i* + 60 to day *i* + 61. In this way, the second iteration (*i* = 2) has a PreHR from day 2 to 61 with a 2-day window spanning days 62 and 63, the third iteration (*i* = 3) has a PreHR from day 3 to 62 with a 2-day window spanning days 63 and 64, and so on. We applied this iterative process to each day throughout the extent of the GPS-collar data for each deer (Fig 1) with an algorithm written in R (S2 File). In effect, we compared each single day’s relocation data to the animal’s most recent 60-day period of space use. We then visually inspected and verified all EHRMs identified by the algorithm within the moving 2-day windows. We utilized a 2-day, rather than a 1-day window, for EHRM detection in order to capture any EHRMs that were initiated by an animal at the end of the first day and that extended into the subsequent day(s); however, we only considered EHRMs for that iteration if they were initiated on day 1 of the 2-day period. We dismissed EHRMs that were initiated on day 2 and re-assessed them during the subsequent iteration, such that the EHRM could then be captured on day 1 of the subsequent 2-day window and viewed in the context of the immediately preceding 60-day home range. As most EHRMs by deer identified in previous studies concluded within 24 hours of commencement [55, 57–59], a 2-day window allowed for the majority of EHRMs to be captured in their entirety without having to manually extend the window, while the 2-day window was also typically short enough to prevent more than one EHRM from being captured at a time. Similar to the parameters of the PreHR duration, the specific length of the moving window is adjustable depending on study species and EHRM characteristics, as the window still shifts by a single day for each iteration. If an EHRM was detected that extended beyond the 2-day window, the 60-day home range iteration was “paused” and we simply extended the 2-day window in which the EHRM was detected until the deer returned to its home range from the EHRM event. In the case of a dispersal event where an individual did not return to the 60-day PreHR, we extended the window until the individual began exhibiting the first indication of fidelity to a new area. In this latter case, we reviewed the movement in the days following the initial EHRM to confirm fidelity to the new area.

### Extra-home range movement definition criteria

To accomplish our second objective of highlighting how variability in EHRM-detection methodologies among previous studies may influence detection rates, inhibit comparisons between studies, and possibly hinder the biological understanding of EHRMs, we performed several analyses in which we applied various detection and definition criteria to our white-tailed deer EHRM dataset previously generated by the Moving-Window method. First, we examined peer-reviewed publications, theses, and dissertations in which EHRM behaviors by white-tailed deer were documented and described. We then narrowed our focus by selecting those studies that used fixed minimum distance and/or duration values to define EHRM behaviors outside of 95% home range contours; we focused on fixed distance and/or duration as these are by far the most common criteria used and most easily replicable, although others do exist (e.g., [57, 73–76]). Studies could contain an EHRM definition using a distance criterion only, a duration criterion only, both distance “AND” duration criteria together, or a distance “OR” duration criterion option. We separately applied each criteria from these studies to the sample dataset of EHRMs generated by our Moving-Window method analysis to compare the number of EHRMs that would have been detected by previously-used EHRM definitions. For the studies which employed the “OR” conjunction in their criteria and where the latter half of the argument was not a fixed distance or duration (e.g., minimum distance OR if ≥50% of daily points were outside of the seasonal home range), then we did not apply the latter half of the criteria to the analysis for computational simplicity.

In our final analysis, to demonstrate the effects of using either the “AND” or “OR” conjunctions as part of an EHRM-definition criterion, we applied fixed distance/duration components (≥1.0 km, ≥12 h outside the 95% contour) to the EHRM dataset generated by our Moving-Window analysis. These values were selected for comparison purposes because they approximate commonly-used criteria values from other studies (e.g., [55, 57–59, 68, 69, 73]). We do not imply preference or biological relevance for these specific distances and durations.

## Results

### Extra-home range movement detection

We used data for 33 collars that fit our criteria for analysis by having collected data for a minimum of 60 days (mean collar duration = 379.6 days, SD = 174.1 days, maximum duration = 577 days). The Fixed-Period EHRM-detection method found that 30 deer collectively exhibited 215 EHRMs extending ≥0.5 km outside of the 95% seasonal home range contours (Table 1). The Moving-Window method found that 32 deer collectively exhibited 320 EHRMs extending ≥0.5 km outside of the 95% home range contours. Neither method detected EHRMs for 1 of our collared females. Extra-home range movements that were not available for detection by both methods were excluded from further analysis (*n* = 17 EHRMs detected by the Fixed-Period method that occurred during the establishment of the first 60-day PreHR of the Moving-Window method and were therefore not available for detection by the latter, and *n =* 18 EHRMs detected by the Moving-Window method that occurred when less than 60 days of data were available in a given season, and thus were not available for detection by the Fixed-Period method). One hundred eighty-two EHRMs were detected by both methods and were available for direct comparison. When detected by the Moving-Window method, mean maximum EHRM distance was 5.6% greater ($\bar{x}$ = 127.0 m, SE = 37.4 m, paired-sample t(181) = 3.40, *p* < 0.001) than when the same movements were detected by the Fixed-Period method. The longest EHRM detected by the Moving-Window method and undetected by the Fixed-Period method extended 4.9 km beyond the home range boundary ($\bar{x}$ = 0.81 km). In contrast, the longest movement detected by the Fixed-Period method and undetected by the Moving-Window method was 1.2 km ($\bar{x}$ = 0.66 km).

**Table 1:** Extra-home range movements detected by Fixed-Period and Moving-Window methods [*n* and %] for white-tailed deer (*Odocoileus virginianus*) in Alabama, USA, 2014–2016.

	EHRM^a^ detection method			
	Fixed-Period		Moving-Window	
Comparison	*n*	%	*n*	%
Total EHRMs detected	215		320	
EHRMs detectable by both methods^b^	198		302	
Unique EHRMs^c^	16	8.1	120	39.7
EHRMs 0.5–1.0 km	14	87.5	105	87.5
EHRMs ≥1.0 km	2	12.5	15	12.5

^a^ Extra-home range movement/s (EHRM/s).

^b^ Not all EHRMs were available for detection by the other method due to EHRMs occurring during the first 60-day PreHR for an individual (not available for the Moving-Window analysis) or occurring if there were less than 60 days of data available in a given season for an individual (not available for the Fixed-Period analysis).

^c^ EHRMs detected by the given method, but not detected by the other method.

### Extra-home range movement definition criteria

We identified 7 previous studies that implemented fixed minimum distance and/or duration criteria for detecting EHRMs by white-tailed deer. The criteria from these studies were applied to the 320 EHRMs identified by our Moving-Window analysis (Table 2). The criteria used by Sullivan et al. [73] matched the criteria used to generate our sample dataset for this portion of our analyses (≥0.5 km outside the 95% contour) and therefore identified 100% of the EHRMs that our sample criteria detected. Aside from this study, the criteria used by other studies identified between 15.6% and 77.5% of the EHRMs from our sample dataset, with a mean detection rate of 45.6%.

**Table 2:** Extra-home range movements [*n* and %] detected when various criteria from white-tailed deer (*Odocoileus virginianus*) studies were applied to our sample dataset from 33 deer in Alabama, USA, 2014–2016.

Criteria	Study reference	Criteria	EHRMs^a^^,^^b^	%
Distance only	Sample dataset	≥0.5 km	320	NA
	Nelson and Mech 1981	>1.6 km	79	24.7
	Kolodzinski et al. 2010^c^	>0.75 km	185	57.8
	Lutz et al. 2016	>1.5 km	83	25.9
	Sullivan et al. 2017^c^	≥0.5 km	320	100.0
Duration only	NA^d^	NA	NA	NA
Distance AND duration	Karns et al. 2011	>0.5 km, ≥6 h	248	77.5
	Olson et al. 2015	≥1.6 km, ≥12 h	56	17.5
	Simoneaux 2015	≥1.6 km, >13 h	50	15.6
Distance OR duration	NA^d^	NA	NA	NA

^a^ Extra-home range movements (EHRMs).

^b^ Number of EHRMs detected from the sample Moving-Window dataset.

^c^ Study applied additional, non-standard "OR" criteria which were not considered here.

^d^ There were no studies documented with these criteria.

**Fig 2:**
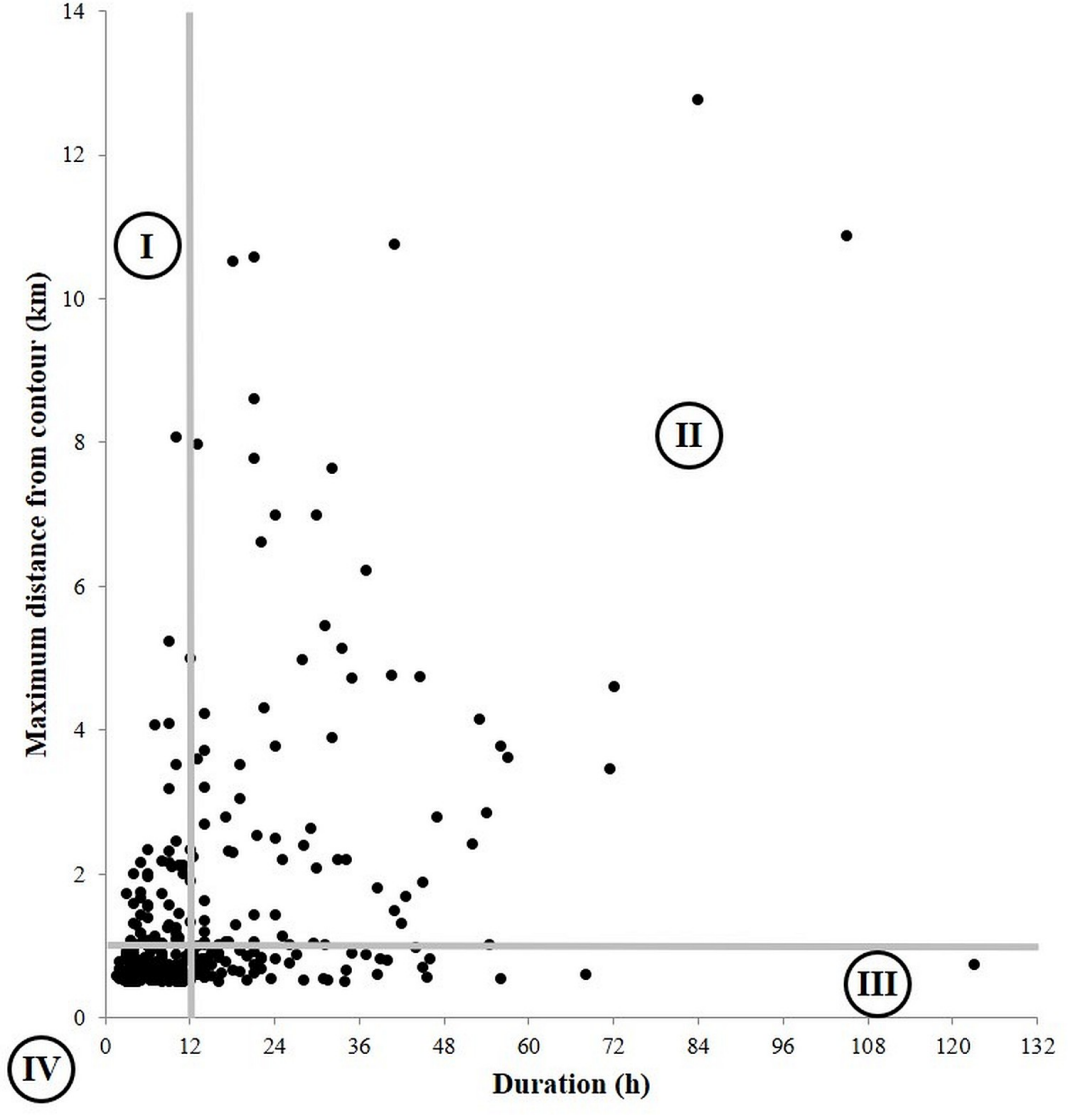
Distance and duration criteria applied to a dataset of 320 extra-home range movements. White-tailed deer extra-home range movement data were collected using a cutoff value of ≥0.5 km outside of 95% home range contours. Sectors of this graph, delineated by vertical and horizontal lines and denoted by the numerals I–IV, indicate portions of this dataset which may be included or excluded from a study where definition criteria include a distance value of ≥1.0 km AND/OR a duration value of ≥12 h.

In our third analysis, when our standardized example criteria (≥1.0 km, ≥12 h) were applied to the sample dataset of white-tailed deer EHRMs (Fig 2), the “distance OR duration” criteria captured the most EHRMs, identifying 85.3% of the total EHRMs (sectors I, II, and III). The “duration only” criterion identified 45.0% of the EHRMs (sectors II and III), followed by the “distance only” criterion, with 40.3% detection (sectors I and II). When the “distance AND duration” criteria were applied, only 24.1% of EHRMs were identified (sector II). Both the “duration only” and the “distance AND duration” criteria omitted 18 movements extending >2.0 km, 4 movements extending >4.0 km, and 1 movement extending >8.0 km outside the 95% home range contour. Likewise, 19 EHRMs lasting >24 h were omitted when using either the “distance only” or the “distance AND duration” criteria, including EHRMs lasting 56 h, 68 h, and 123 h.

## Discussion

### Extra-home range movement detection

The Fixed-Period approach for identifying EHRMs in animal movement studies typically involves constructing a temporally-fixed, seasonal or annual home range for an individual and then examining GPS locations recorded during that fixed period for any long-distance movements extending outside of that home range (e.g., [61, 68, 77–79]). The Fixed-Period approach did not detect a substantial proportion of EHRMs (39.7%) that were identified by the Moving-Window method in our study and missed movements that extended up to nearly 5 km outside the home range boundary. These undetected EHRMs were likely missed as a result of one of three scenarios: 1) the EHRMs led to direct changes in the animal’s space use following the EHRM to incorporate that area visited into its home range; 2) the extent of the EHRMs themselves influenced the utilization distribution modeled by the home range estimator; or 3) subsequent movements unrelated to the EHRM (such as a subtle seasonal shift in space use) may have occurred and masked the prior-occurring EHRM. All three scenarios, and scenario 1 in particular, have the potential to yield insights regarding biologically relevant EHRMs and our understanding of EHRM motivation and how EHRMs relate to an animal’s future space use [80]. In contrast, only 8.1% of Fixed-Period EHRMs went undetected by the Moving-Window method. These 16 missed movements were all of shorter distance (approximately 1 km or less outside the home range boundary and 0.66 km on average) and were likely missed as a result of minor differences in home range boundary contours modelled by the Fixed-Period and the Moving-Window methods that allowed the EHRM to fall on either side of the 0.5 km threshold and therefore be eligible for detection by one method but not the other. This is possibly the case, as well, for some of the 120 unique EHRMs that were detected by the Moving-Window method but missed by the Fixed-Period method; however, the extreme distances characteristic of many of the EHRMs missed by the Fixed-Period but detected by the Moving-Window method suggest that a large portion of these missed EHRMs were likely the result of one of the three previously mentioned scenarios. Returning to the questions we previously posed and reframed for EHRM detection (“What is the animal’s home range over the period from time *X* up to time *Y*?”, and “At time *Y*, did the animal leave the home range which existed from time *X* up to time *Y*?”), approaching EHRM detection by accounting for an animal’s prior movements helps avoid missing important EHRMs that would be masked when searching for EHRMs in the context of a larger, fixed-period home range that incorporates not only prior, but future, movements to define the animal’s typical area of space use.

Mean maximum EHRM distance was 5.6% greater than when the same movements were detected by the Fixed-Period method. This suggests that when some EHRMs are detected by the Fixed-Period method, shifts in subsequent space use and/or the use of the EHRM relocation points in constructing the seasonal home range are also likely masking a portion of the same, longer EHRM that was detected by the Moving-Window method. Again, we suggest that this incomplete picture of EHRM activity—either when EHRMs are partially masked or when they are wholly obscured by a fixed-period home range that includes post-EHRM movement data—has been one of the primary obstacles in understanding EHRM behaviors in general. We believe this is especially true regarding white-tailed deer and roe deer, due to the reliance on seasonal home ranges in these studies (e.g., [55–62, 77]). In the 21^st^ century, several researchers have rejected previously proposed theories regarding EHRMs motivations, yet have been unable to offer any alternative hypotheses for why they occur [56, 59, 69]. This will likely remain an issue as long as EHRMs, and the subsequent space use following an EHRM, are used to construct the home ranges that are themselves used for detecting the EHRMs during that time period.

A primary reason that fixed-period, seasonal or annual home ranges have been used in past studies for detecting EHRMs is that they theoretically provide adequate representation of an animal’s normal space use over an extended period when behavior should remain relatively constant [27, 81] and therefore would be useful for detecting deviations from this normal space use (EHRMs). However, in some cases, this “adequate representation of space use” has not actually been established by the time an EHRM occurs. When an EHRM occurs during the early part of a season (e.g., 17 days after the start of a 120-day “Winter” seasonal home range period), the seasonal home range would be primarily comprised of movement data occurring after the EHRM event, rather than before the event occurred. This would defeat the purpose of using an established pattern of movement to detect deviations from this pattern, as most of the data used to establish the pattern would be generated post-EHRM. We argue that if the Fixed-Period method is employed in future studies for EHRM detection and analysis, then EHRMs detected during the early portion of each given season should be omitted from the results since a “typical” area of space use could not have been constructed by that point (though they may still represent biologically valid EHRMs). In our comparison of detection methods, 17 EHRMs detected by the Fixed-Period method were omitted from the Moving-Window method because they occurred during the first 60-day PreHR period of an individual deer, and thus were not available for detection. However, given our argument that EHRMs should not be considered for analysis unless an adequate area of prior, typical space use has been modelled with relocation data, we do not consider this a limitation of the Moving-Window method, as many of those EHRMs would then be omitted from the Fixed-Period method as well.

Implementation of the Fixed-Period method will likely result in researchers censoring large quantities of relocation data, as full seasons are often not captured at the beginning or end of each deer’s dataset, and incomplete seasons should not be used for EHRM detection if a true (i.e., “complete”), seasonal home range is desired to establish an animal’s typical space use. In our test of the Fixed-Period method, we accepted datasets for use if at least 60 days of data existed within a season in order to have adequate amounts of data for comparison; however, this does not align with the true concept of a complete, seasonal home range. In our analysis, 29.3% of EHRMs detected by the Fixed-Period method were detected during incomplete seasons—that is, seasons where relocation data did not span the entire length of the season due to animal collar deployment date as well as due to the termination date of the data collection period (e.g., collar drop-off date, collar malfunction, or animal death). If we had limited our analysis to complete seasons only (119–122 days), all of these EHRMs would have been excluded from our analysis. While biologically valid EHRMs may occur during these partial seasons of data collection, the use of those data would be inconsistent with researchers’ intentions of defining a complete seasonal period of space use for EHRM detection. Though an ideal research scenario for the Fixed-Period method would allow all wildlife capture and subsequent relocation data to initiate and terminate precisely at the beginning or end of a given season, or would be conducted with tracking collars capable of both extremely fine-scale data collection while avoiding battery life limitations and therefore could afford researchers the flexibility to censor partial seasons from much larger datasets, this is not feasible for most researchers and these limitations are common artifacts of wildlife research.

In our analysis, we determined that a 60-day PreHR window was an appropriate period to implement for white-tailed deer when examining the overall occurrence of EHRMs by the species given our climate, study parameters, and species behavior, but this period should be adjusted by taxa and study area for the amount of time needed to define a typical area of use and may be adjusted depending on the biological question at hand. The Moving-Window approach can be implemented with a PreHR period of almost any duration. For example, when attempting to detect EHRMs by highly mobile species or by those species with large home ranges such as wolverines (*Gulo gulo*) [82], African wild dogs (*Lycaon pictus*) [83], or griffon vultures (*Gyps fulvus*) [84], a much longer temporal period may be desired to accurately establish a typical area of space use. In contrast, where species have clearly defined, defended, or confined home range boundaries, such as in red squirrels (*Tamiasciurus hudsonicus*) [85] or hooded warblers (*Setophaga citrina*) [43], a shorter-duration period may be adequate for defining the boundaries of an individual’s typical space use. Researchers interested in examining disease spread outside of an “endemic” area for an individual animal may desire to establish a PreHR extending over a larger period of time to better convey a typical space use area, while investigations into animal movements in ecosystems with dramatic fluctuations in resources or rapidly changing environmental conditions may desire a much shorter PreHR (assuming a relocation fix rate fine enough to generate ample data points for home range modelling over a short period). Research conducted in areas with more dramatic climatic and resource shifts and attempting to answer questions regarding an animal’s space use in relation to seasonal changes may dictate a PreHR specific to typical season length, or may dictate a PreHR that extends across an entire 12-month period to capture inter-seasonal changes. It should also be noted that no EHRMs would be available for detection until PreHR + 1 days after the start of data collection, which is a major factor to consider if extremely long PreHR periods are desired (such as when the PreHR duration is 120, 240, or 365 days). While a Fixed-Period approach to EHRMs utilizing a season with a duration of 120 days may appear similar to a Moving-Window approach with a PreHR duration of 120 days, we emphasize that the relationship between the EHRM and preceding and subsequent space use in these two methods is inherently different and has substantial implications for EHRM detection.

Other adjustments to our Moving-Window method can, and should, be made as needed, especially involving instances of migration events or dispersal activity. When instances of juvenile dispersal were observed in our study, we postponed our Moving-Window algorithm until 60 days after the dispersal event concluded. This was done to allow individual animals time to establish patterns of space use on the new landscape. Though migratory events by deer are not typically known to occur in the southeastern United States where our study took place [86], postponing the start of the EHRM detection process where the species of interest may be migratory until *t* days post-completion of the migration event, (where *t* is the number of days used to establish the PreHR period) is recommended.

One aspect of the Fixed-Period method of EHRM detection that may be viewed as an advantage in answering some research questions is that it only identifies EHRMs which do not directly result in, or are not followed by, subsequent (related or unrelated) changes in space-use for the animal and that occur distinctly outside of the animal’s home range for the duration of the given fixed-period (such as season). However, we argue that the Fixed-Period approach still offers an incomplete picture in this regard, particularly when the EHRM occurs toward the end of the temporal frame of the fixed-period (such as at the end of the season). In this case, little data would be available at the end of this fixed-period for a researcher to be able to determine if there was a shift in space use following the EHRM or not, and could lead to erroneous conclusions regarding subsequent space use after an EHRM. We highlight that the Moving-Window approach, as a stand-alone analytical tool, is capable of detecting both EHRMs that may, or may not, result in or are followed by subsequent changes in space use. However, by itself, this approach does not provide researchers with a method of differentiating between these two scenarios. We strongly recommend that all EHRM studies pursue the question of whether or not a change in space use ensued following an EHRM to gain a better understanding of how environmental conditions and associated resources on the landscape (food, water, minerals, mates, safety, etc.) may be associated with these EHRM behaviors. This can be accomplished simply by adding to the same Moving-Window algorithm in R, pairing each identified EHRM with a post-EHRM home range model of the animal’s space use over a desired interval [80].

While our analysis focused specifically on the introduction of the Moving-Window method for detecting non-permanent EHRMs by a medium-sized ungulate, the plasticity of the Moving-Window method in regards to the length of time used to establish home range fidelity or typical area of use (the PreHR) and the duration of the moving-window itself allow this approach to be applied to a broad range of taxa that exhibit some degree of site fidelity over a period of time [54]. This approach can also be utilized or incorporated into other movement models in order to investigate dispersal, migration, or nomadic movement events or periods exhibited by these species [87–90], particularly in identifying the onset of these movements and preceding irregularities in movement patterns. This is of particular importance as researchers continue to seek to understand the mechanisms by which animal diseases and parasites spread across the landscape [91–94]. A similar comparative analysis between a fixed-period approach and a moving-window approach to avian home range movements is presented by Messinger et al. [95] for the purpose of monitoring continuous changes and variability in environmental conditions and their influences on animal behavior. Like ours, this analysis shows the value of reassessing commonly-used methodologies and highlights instances where an iterative, moving-window may reveal additional insights into animal behavior that traditional fixed-period approaches may not.

### Extra-home range movement definition criteria

In discussing the concept of territoriality in vertebrates, Maher and Lott ([96]:1581), stated that, “The potential for instructive comparisons is high, but the comparative approach requires common terminology. Vague or implicit definitions of spacing systems undermine the rigour of comparisons […] Workers pursuing research in this area can enhance their contribution by using clear conceptual and operational definitions of territoriality, making them explicit at the outset.” Similarly, Progulske and Baskett ([97]:189) remarked that “Comparisons [of white-tailed deer movements] are difficult because of the differing techniques of investigation and expressions of results.” We found these arguments to also be true concerning the criteria and terminology used to describe and define EHRMs by white-tailed deer in scientific literature. We suggest that the various descriptors and definitions, while still providing insight into animal behavior, hinder study replication and preclude those “instructive comparisons” between studies. To address this, we suggest 4 guidelines for future studies investigating EHRM behaviors by all species. We argue that future studies of this nature should 1) use clear, unifying terminology, 2) clearly present the definition criteria used for detecting these movements as well as the justification behind using those criteria 3) apply definition criteria suited to the species or taxonomic group of interest, and 4) tailor definition criteria to the biological question being asked.

#### Guideline 1—Terminology

Among literature which document some degree of EHRM behaviors, at least 5 different terms have been used to describe these movements. The term “excursion” has been the most widely-used term characterizing EHRMs, though the terms “trips,” “forays,” “sallies,” and “exploratory movements” have also been used to document instances of irregular, infrequent, long-distance movement behaviors (e.g., [36, 40–42, 98, 99]). In one instance, both “excursions” and “exploratory sallies” were used to describe movements by heteromyid rodents [41]. However, the majority of studies use one of these 5 respective terms exclusively, meaning that a keyword search of the literature including only one of the terms would return a substantially limited set of results. While the first 4 terms listed are close synonyms, the fifth term—“exploratory movement”—implies a specific motivation: that of investigating, wandering, or searching. Not only does this term exclude from the onset the possibility that particular EHRMs may be undertaken for a variety of motivations and assume that all EHRMs detected by a study occur for the same purpose, it also implies that the detection of an EHRM itself without further investigation can reveal the purpose of an animal’s movement. While some EHRMs may indeed be exploratory in nature, no evidence has been presented to support this as an exclusive motivation, at least among ungulates [55, 59, 69]. To reach a clearer understanding of the behaviors and motivations that are manifested in these movements, we propose an operational definition of the term “extra-home range movement” to mean “any non-permanent, long-distance movement outside of a typical area of use.” For further clarification, we suggest that “non-permanent” in this context indicates that the animal returned from the EHRM, for any duration, to the home range which was modelled as the animal’s typical area of use prior to the initiation of the EHRM; that “long-distance movement”, while the actual scale will clearly vary depending on taxa and could also vary depending on factors such as climate and study site, is a minimum distance or spatial segregation value separating outlying strings of sequential relocation points from daily movements within the animal’s home range; and that “outside of a typical area of use” refers to the modelled boundary of spatially and temporally clustered points that are used to define an animal’s home range. We also suggest that future studies examining non-migratory, “excursive-type behaviors” utilize the term “extra-home range movement (EHRM)” when referring to these behaviors. However, it should also be noted that use of the term “extra-home range movement” does not necessarily imply that all of these movements should be viewed in the same context or held to the same criteria (see guidelines 2, 3, and 4 below).

#### Guideline 2—Criteria and justification

No two white-tailed deer studies examined here employed the same criteria for defining and distinguishing EHRM behaviors from the typical area of use. This means that previous instances of study-design replication and reproduction (“the cornerstone of science” [100]) are likely non-existent on the topic of EHRM behaviors and that future reproducibility will be subject to selecting from a large pool of often inadequate methodologies and criteria. When criteria from those studies were applied to our sample EHRM dataset, the spectrum of EHRM-definition criteria led to dramatically varied detection rates of EHRMs and detection of distinct, but incomplete, subsets of the sample data. Up to 85% of the EHRMs in our sample dataset were missed by these different detection criteria; however, all but 1 of these studies used the same term—“excursion”—to describe the movements being measured. Further variations in detection criteria have been reported in additional studies focused on EHRMs by white-tailed deer. Some of these (e.g., [56, 76, 101]) calculated EHRM distances from home range or activity centers, rather than contours, and Basinger (2013) [74] measured EHRM distances from 90%, rather than 95%, home range contours. Additionally, when a single, example set of criteria were applied to our dataset, a wide range of EHRM detection rates were obtained (24–85%), simply by interchanging the “AND/OR” conjunctions between terms. When certain criteria were used, several extremely long-distance or long-duration movements would have been excluded. Those movements were arguably intentional movements outside of a typical area of use, not random movements along the home range periphery. The omission of these movements risks missing biologically important behaviors. All of these examples highlight the need for caution when comparing what appear to be similar assessments of EHRM behaviors from previous studies, as well as the need for complete reporting of all criteria used to define these movements.

Furthermore, when a set of criteria for determining EHRMs is selected, a detailed biological justification explaining the selection of those criteria should be included in the methodology. Researchers have struggled to use mathematical models and parameters to identify and separate biologically different movements when it comes to EHRMs and other daily movements, and this will likely continue to be the case until more advanced spatial segregation methods are developed and become available. However, without advanced knowledge of each individual animal’s cognitive process and decision-making, complete understanding of what separates one movement behavior from another is likely not possible. In lieu of this, mathematically-derived values are applied to movement data to detect these differences. In instances where fixed distances and durations are applied as definition criteria to distinguish between EHRMs and other, subtler, movements along the home range periphery but that fall outside the 95% home range contour, these values (e.g., 1.0 km, 1.6 km, 6 h, 12 h, 24 h) are, however, often arbitrary and are presented without any attempt at biological justification. Additionally, the use of individual-based movement metrics (e.g., >2x the standard deviation of center-to-fix distance [76]) are arbitrary themselves and usually do not adequately measure all instances of biologically (or statistically) founded spatial outliers. Most previous studies fail to provide justification for their methodology selection, likely because there is no clear biological support for these values, especially in choosing one fixed value over another. It might be argued that the fixed value of 0.5 km used in this analysis of detection methodology is also arbitrary; however, we selected this value based on a biologically-relevant scale to white-tailed deer movements (e.g., [55–57, 59]) and as a conservative threshold among past EHRM studies to minimize the potential of missing intentional EHRMs, while at the same time avoiding unnecessary examination of each relocation falling outside a 95% home range contour that occurred during occasional movements along a home range periphery. We suggest that future analyses intending to examine EHRMs should clearly document all analysis criteria and definitions to allow for critique, reproducibility, and comparison between studies [102]. It is also critical that considerable attention and documentation be given to the justifications for particular criteria selection and utilization. We advocate that researchers seek to balance this specific justification with the desire for standardization among EHRM studies, and we suggest the need for a general consensus among researchers on a suite of EHRM definition criteria, at least when these movements are viewed in a similar context, similar environmental conditions, or within a particular taxon (see guidelines 3 and 4 below). Arriving at a consensus may require exploring and/or improving alternative means of identifying spatially segregated and abnormal movements such as cluster analyses and spatial outlier detection methods (e.g., [103–105]).

#### Guideline 3—Species/taxa of interest

Though our analysis focused on examples of EHRM behaviors found in published studies on white-tailed deer, movements of a similar nature have been documented and explored in the context of ungulate species such as roe deer [60, 61, 77, 106, 107] and red deer [62, 108–110], as well as in other mammalian and avian species, herpetofauna, and ichthyofauna [31–49]. It is likely that many of these species exhibit EHRMs for biologically similar reasons, particularly concerning resource acquisition (i.e., mate, mineral, food) or exploration (which may also include pre-dispersal and final dispersal movements by juveniles). However, a single, particular criterion may not be appropriate across all taxa. We define EHRMs as “long-distance movements,” but distance here should be relative to the typical scale of movement for the species in question. Similarly, it may not be appropriate to compare all behaviors characterized as EHRMs, even within taxa, using the same criteria. White-tailed deer, roe deer, and red deer all employ different space use tactics (e.g., territoriality) and breeding systems (e.g., harems) throughout the year [111–113]. Though each of these deer species has been shown to exhibit EHRMs, it is possible that different factors drive these behaviors in each species. Currently, EHRM studies concerning roe deer and red deer [60–62, 77, 106] have limited their analyses to the breeding season only, and thus, a thorough review of other potential factors driving EHRMs has not yet been conducted. Furthermore, space use within and around the home range of a territorial species versus a non-territorial species is likely to be inherently different. Short-distance movements outside of the defined home range of a territorial species could be more biologically meaningful than short- or even long-distance movements outside the 95% home range contour of a species that does not exhibit territorial behavior. Therefore, even if individuals of each species were exhibiting EHRM behaviors for the same reason (such as for inbreeding-avoidance), utilization of the same fixed-distance criteria to define these behaviors would be inappropriate and ineffective.

#### Guideline 4—Question of interest

At the heart of the confusion among EHRM studies is the following question: are researchers trying to define EHRMs based on the motivations behind the behavior (e.g., breeding-related EHRMs) or based on what the movements themselves look like across a landscape (e.g., straight-line return movements extending beyond a particular distance and duration)? This may appear like a trivial case of semantics; however, both approaches have regularly been employed and we argue this further complicates the topic of EHRMs. Researchers investigating EHRMs during the breeding season may desire to understand how highly tortuous movements by males just outside the periphery of home ranges relate to female receptivity and conception. In contrast, researchers investigating the spread of chronic wasting disease from localized hot zones to outlying counties may only be interested in the occurrence and frequency of extremely long-distance, EHRMs by deer. Still others may be solely interested in determining the motivations behind why animals leave their home ranges on these movements. We suggest that if EHRMs are to be documented and discussed, researchers should clearly state why these movements are of interest and in what context these movements are to be viewed. This will aid in providing justification for the criteria used to describe and identify EHRM behaviors, as well as justifying why the selected approach is appropriate for the taxa and question of interest.

## Supporting information

S1 File Fixed-Period algorithm. The algorithm written in R and used for detecting extra-home range movements outside of seasonal home ranges in this analysis.
(R) Click here for additional data file.

S2 File Moving-Window algorithm. The algorithm written in R and used for detecting extra-home range movements within a 2-day moving window following a 60-day home range period in this analysis.
(R) Click here for additional data file.
